# Development of the Gambling Disorder Identification Test: Results from an international Delphi and consensus process

**DOI:** 10.1002/mpr.1865

**Published:** 2020-11-21

**Authors:** Olof Molander, Rachel Volberg, Viktor Månsson, Kristina Sundqvist, Peter Wennberg, Anne H. Berman

**Affiliations:** ^1^ Department of Clinical Neuroscience Center for Psychiatry Research Karolinska Institutet Solna Sweden; ^2^ Stockholm Region Health Services Stockholm Sweden; ^3^ School of Public Health and Health Sciences University of Massachusetts Amherst Massachusetts USA; ^4^ Department of Psychology Stockholm University Stockholm Sweden; ^5^ Department of Public Health Sciences Stockholm University Stockholm Sweden; ^6^ Department of Global Public Health Karolinska Institutet Solna Sweden; ^7^ Department of Psychology Uppsala University Uppsala Sweden

**Keywords:** Delphi, gambling disorder, Gambling Disorder Identification Test (GDIT), problem gambling, psychometric development

## Abstract

**Objectives:**

Diverse instruments are used to measure problem gambling and Gambling Disorder intervention outcomes. The 2004 Banff consensus agreement proposed necessary features for reporting gambling treatment efficacy. To address the challenge of including these features in a single instrument, a process was initiated to develop the Gambling Disorder Identification Test (GDIT), as an instrument analogous to the Alcohol Use Disorders Identification Test and the Drug Use Disorders Identification Test.

**Methods:**

Gambling experts from 10 countries participated in an international two‐round Delphi (*n* = 61; *n* = 30), rating 30 items proposed for inclusion in the GDIT. Gambling researchers and clinicians from several countries participated in three consensus meetings (*n* = 10; *n* = 4; *n* = 3). User feedback was obtained from individuals with experience of problem gambling (*n* = 12) and from treatment‐seekers with Gambling Disorder (*n* = 8).

**Results:**

Ten items fulfilled Delphi consensus criteria for inclusion in the GDIT (*M* ≥ 7 on a scale of 1–9 in the second round). Item‐related issues were addressed, and four more items were added to conform to the Banff agreement recommendations, yielding a final draft version of the GDIT with 14 items in three domains: gambling behavior, gambling symptoms and negative consequences.

**Conclusions:**

This study established preliminary construct and face validity for the GDIT.

## 1 INTRODUCTION

Problem gambling (PG) is an international growing concern for public health authorities and health care systems (Calado & Griffiths, 2016) and is defined as “excessive gambling behavior that creates negative consequences for the gambler, others in his/her social network, and for the community” (Blaszczynski & Nower, 2002). From a public health perspective, identifying PG is a challenge met to some extent by self‐report assessment, such as the widely‐used Problem Gambling Severity Index (PGSI; Ferris & Wynne, 2001). From a clinical perspective, the diagnostic criteria in the latest edition of the Diagnostic and Statistical Manual of Mental Disorders (DSM‐5) were revised in 2013 and labeled Gambling Disorder (GD), with three levels of symptom severity (American Psychiatric Association, 2013). At the same time, gambling was classified together with substance use disorders, covering alcohol and drug use, which have long been the focus of extensive research on assessment, trajectories of use, and treatment outcomes.

A major persistent issue has been how to measure PG and GD (Caler, Garcia, & Nower, 2016; Dowling et al., 2017; Pickering, Keen, Entwistle, & Blaszczynski, 2017). In an effort to examine the global prevalence of PG across countries and time, Williams, Volberg, and Stevens (2012) compared 202 studies conducted between 1975 and 2012. The standardized past year rate of PG ranged from 0.5% to 7.6% internationally over time, with an average rate across all countries of 2.3%. Several methodological issues affecting PG prevalence were reported, such as different time frames used to assess PG, variations in the administration of measures or differing scoring thresholds for PG from the same measure used in different studies. The extent to which existing measures are valid proxies for the different severity levels covered by the GD diagnosis remains unclear, particularly since the introduction of the DSM‐5 diagnostic criteria in 2013.

To address the overarching issue of variations of measures in gambling treatment studies, an expert committee of gambling researchers convened in 2004 at the Alberta Gambling Research Institute's 3rd Annual Conference (Walker et al., 2006), an annual independent gambling conference in Banff, Canada. The result, known as the Banff consensus agreement, was a major step forward in the conceptualization of a framework for minimal features of treatment outcome measures. The Banff framework stipulates three domains: (1) measures of gambling behavior (net expenditure each month, the frequency in days per month when gambling takes place, and time spent thinking about or engaged in the pursuit of gambling each month); (2) measures of the harms caused by gambling (personal health, relationships, financial and legal); and (3) measures of the proposed mechanism of change in a specific treatment. At the time of the Banff consensus, it was clear that one obstacle to its realization was the lack of existing gambling measures that fully complied with it (Walker et al., 2006).

A recent systematic review (Pickering et al., 2017) concluded that most gambling studies failed to fulfill the measurement guidelines outlined by Walker et al. (2006). Furthermore, a comprehensive analysis of existing gambling measures (Molander et al., 2019) identified limitations in terms of content validity. Categorization of all items in 47 different gambling measures showed that they targeted a wide range of constructs, such as PG symptoms and urges, gambling behavior, monetary aspects, negative consequences of gambling, cognitive distortions, motivation and self‐efficacy (Molander et al., 2019). Despite the passage of time, it was still the case that no measure seemed to adequately fulfill the recommendations in the Banff consensus (Walker et al., 2006). An additional limitation was that few measures were validated in relation to the new DSM‐5 criteria for GD (Molander et al., 2019). Even more recently, a systematic review identified 31 different screening instruments from 60 studies, finding that only 3 instruments had been validated against the DSM‐5 criteria for GD (Otto et al., 2020).

In order to redress this situation, we initiated a process to develop the Gambling Disorder Identification Test (GDIT), as an instrument measuring the frequency of gambling behavior as well as related symptoms and consequences, analogous to the Alcohol Use Disorders Identification Test (AUDIT; Saunders, Aasland, Babor, de La Fuente, & Grant, 1993) and the Drug Use Disorders Identification Test (DUDIT; Berman, Bergman, Palmstierna, & Schlyter, 2005). Using the AUDIT and DUDIT as a point of reference for this development process has several potential advantages. First, the AUDIT and the DUDIT content (substance use behaviors, dependence symptoms and negative consequences) corresponds to the first two domains of gambling behavior, and problems caused by gambling, recommended in the Banff consensus (Walker et al., 2006). We did not include the third Banff domain, items measuring processes of change, as such measures are treatment specific and need to be tailored to a range of possible theoretical assumptions. Secondly, the AUDIT and the DUDIT are widely used internationally to identify and assess problematic substance use within health care‐ and social service systems, as well as public health agencies (for reviews see Hildebrand, 2015; Reinert & Allen, 2002). Developing a measure for gambling similar to the AUDIT and the DUDIT is compatible with the DSM‐5 decision to label gambling as an addictive behavior, and more easily facilitate implementation of screening procedures for PG. Third, the AUDIT and the DUDIT use frequency‐based categories asking the respondent to state how often substance use behavior as well as dependence symptoms and consequences occur, for example “Never, Less than once a month, Every month, Every week, Daily or almost every day”. This is an advantage compared to existing gambling measures using dichotomous “Yes/No” (e.g., the NORC Diagnostic Screen for Gambling Problems [NODS; D. C. Hodgins, 2004]); or vaguely stated verbal item responses, for example “Not at all, Rarely, Sometimes, Often” in the PGSI (Ferris & Wynne, 2001). Developing a gambling measure using specified frequency‐based behavioral categories will enable clearer measurement procedures (e.g., De Vet, Terwee, Mokkink, & Knol, 2011) as well as possibly facilitate comparisons between problematic substance use and PG behavior.

The GDIT development process has included four steps, generally aligned with the instrument development steps outlined Gehlbach and Brinkworth (2011): (1) identification of items that might be eligible for the GDIT from a pool of existing gambling measures; (2) presentation of proposed items for evaluation by invited experts in gambling research, clinical practice and treatment training, through an online Delphi process and subsequent consensus meetings to determine included items and formulate new items as necessary; (3) pilot testing of a draft version of the GDIT for face validity in a small group of participants with self‐experience of PG (*n* = 12), as well as preliminary psychometric properties in a small group of treatment‐seeking participants with PG or GD (*n* = 8); and (4) evaluation of the psychometric properties of the final GDIT measure in relation to existing instruments and semi‐structured interviews assessing the DSM‐5 criteria for GD, among individuals with PG or GD as well as non‐problematic recreational gambling behaviors (sample target *n* = 600). The first, second and third steps have been completed and the fourth step is now underway. The first step, with identification and content‐based categorization of 583 unique items from 47 existing gambling measures, has been described in a published research protocol (Molander et al., 2019). This first step also involved selection of 30 possible items eligible for inclusion in the GDIT, based on inter‐rater agreement on items relevant for the proposed GDIT domains, previous psychometric findings regarding PG (Chamberlain, Stochl, Redden, Odlaug, & Grant, 2017; Stinchfield et al., 2016; Volberg & Williams, 2011) as well as the Banff consensus recommendations (Walker et al., 2006).

Our aim in this article is to describe steps two and three, showing how a consensus was reached regarding a specific set of items, and yielding a testable draft version of the GDIT. The consensus process built on prioritizing item domains recommended in the Banff agreement, with international input from a Delphi process with an ensuing consensus procedure. The research questions in this study are:

1. Which items should have the highest priority for inclusion in the GDIT?
2. What possible problematic issues emerged concerning the prioritized items?
3. How might problematic issues among the prioritized items be addressed?
4. Which additional items would need to be included in the first GDIT version, in order to fully comply with the Banff consensus agreement recommendation?

## 2 METHOD

**FIGURE 1:**
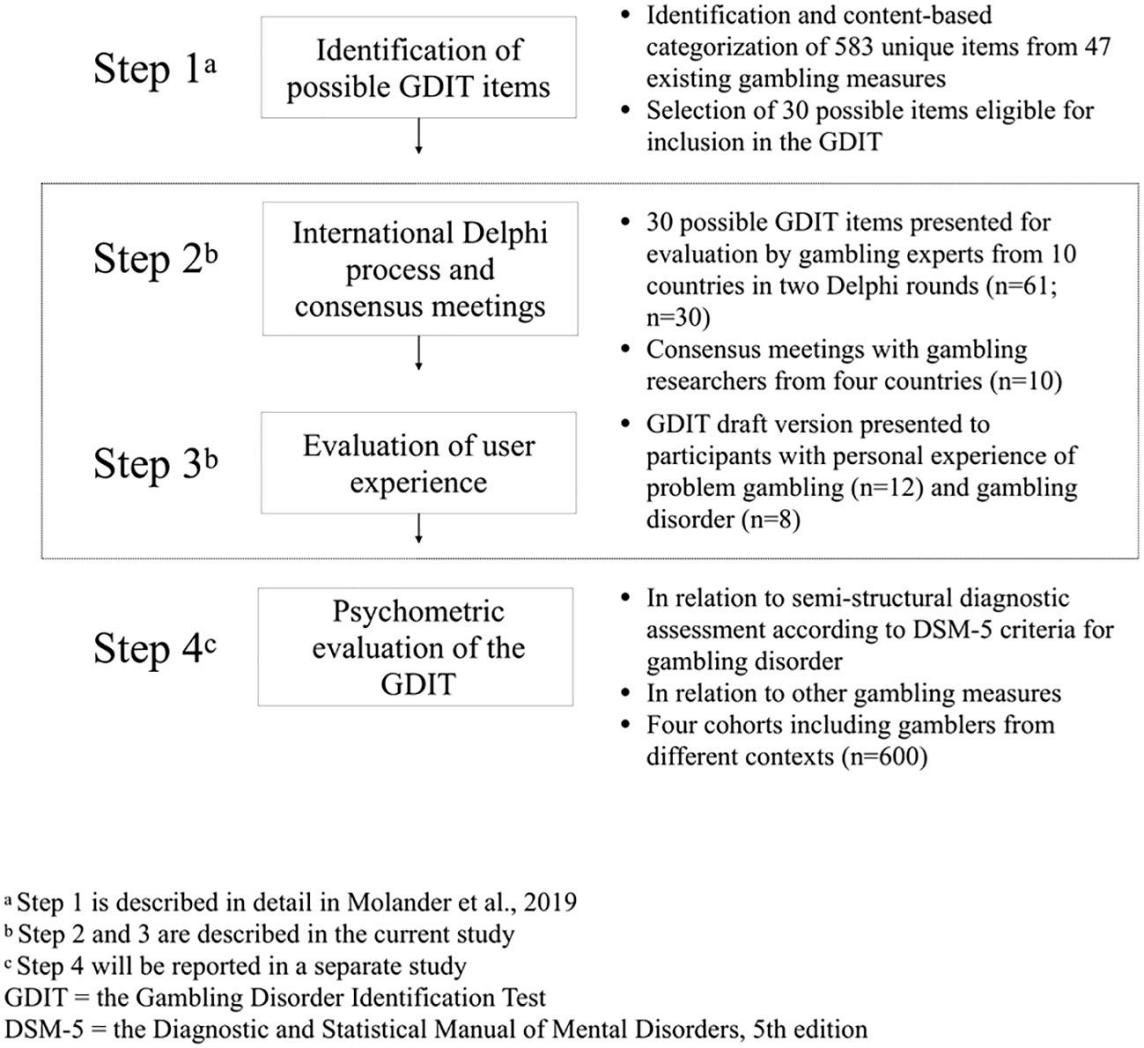
Development of the Gambling Disorder Identification Test (GDIT), in four steps

The methodology used in the GDIT development process has been described elsewhere (Molander et al., 2019). Briefly, the process builds on several interdependent stages (see Figure 1), where the recommendations from the Banff consensus were given priority beyond the Delphi results.

### 2.1 Delphi survey rounds

An online international Delphi survey was launched with a presentation of the 30 items eligible for inclusion in the GDIT that were identified in step one (Molander et al., 2019). Using snowball sampling, we invited an extensive range of expert stakeholders to participate, aiming to include as many relevant stakeholders as possible. The invitation was sent to (1) all authors of the Banff consensus (Walker et al., 2006), (2) corresponding authors of articles reporting previous psychometric findings as well as reviews of gambling measures identified in our preparatory study (Caler et al., 2016; Chamberlain et al., 2017; Dowling et al., 2017; Stinchfield et al., 2016; Volberg & Williams, 2011), (3) first and last authors of reports and articles on gambling measures (see Molander et al., 2019) as well as (4) authors of reports on randomized trials evaluating interventions for PG and GD, published in systematic trials (Cowlishaw et al., 2012; Pallesen, Mitsem, Kvale, Johnsen, & Molde, 2005; Petry, Ginley, & Rash, 2017) identified in our preparatory study (Molander et al., 2019). We also invited all presenters at the Alberta Gambling Research Institute's 17th Annual Conference, 2018, members of the ongoing six‐year research program on Responding to and Reducing Gambling Problem Studies, as well as members of the Swedish Gambling Research Network, a network convening Swedish researchers, clinicians and treatment trainers in the gambling field. Invitations to participate in the first round of the Delphi process were sent by e‐mail on March 16th, 2018 to 170 stakeholders, including the authors of this article. Some stakeholders were sent invitations to multiple email addresses that were identified, for example, via published articles or academic institutions. Stakeholders who completed the first round of the Delphi within two weeks were sent an invitation to participate in the second round. For each round, a single e‐mail reminder was sent after one week to stakeholders who did not complete the questionnaire.

The 30 items were presented in the first Delphi round with a rationale for possible inclusion in the final GDIT draft. An example of the text presented is as follows:

Item 8. How often have you gambled to win back money you lost, the past 12 months? Rationale: “Chasing losses” is a key dependence symptom in the diagnostic criteria of Gambling Disorder. Denis, Fatséas, and Auriacombe (2012) found that “chasing losses” in addition to three other DSM‐ IV criteria (repeated unsuccessful efforts to stop, lies, and jeopardized/lost relationships/job) best discriminated pathological‐ and non‐pathological gamblers. In a later study of DSM‐5 criteria. Chamberlain, Stochl, Redden, Odlaug and Grant (Chamberlain et al., 2017) found that “the main diagnostic item serving to discriminate recreational from problem gamblers was endorsement of chasing losses”.

Participants were asked to rate the importance of each item for inclusion in the GDIT on a scale from 1 to 9, where scores of 1–3 were classed as “not important for inclusion,” 4–6 were classed as “important but not critical,” and 7–9 were classed as “critical for inclusion” (see Guyatt et al., 2011). In addition, stakeholders were offered space for optional comments on each item regarding possible problematic issues, such as psychometric relevance and accuracy, semantic item structure and content of multiple‐choice alternatives. For the second Delphi round, the results from the first Delphi round were compiled and item ratings as well as all stakeholder comments for each item were presented. The respondents were asked to reflect on the results and to rate and comment on each item again. The consensus criterion regarding the importance of including an item in the GDIT was set to *M* ≥ 7 for each item in the second survey round; in view of the lack of guidelines for Delphi consensus criteria, we chose to set the consensus criterion to include items rated in the top third of the rating scale. The results of Delphi rounds 1 and 2 were presented in three following consensus meetings with gambling researchers, where each item with its response categories was reviewed and discussed. This yielded a final selection of items, based upon (1) the recommended features of gambling measures in the Banff consensus (Walker et al., 2006), and (2) the consensus criteria in the expert Delphi.

### 2.2 Consensus procedure

The results from both Delphi rounds were first presented at a consensus meeting on April 14, 2018 at the Alberta Gambling Research Institute's 17th Annual Conference, in Banff, Alberta, Canada (Molander et al., 2018). Participants in the consensus meeting were 10 gambling and addiction researchers from five countries, eight of whom had participated in the Delphi, and two of whom were recruited on site; all agreed to participate in the consensus meeting. Two following consensus meetings with a sub‐group of four gambling researchers from two countries were held in Stockholm, at Karolinska Institutet, on May 8, and May 30, 2018. The purpose of all consensus meetings was to resolve issues in items through discussion and consensus decisions, in order to arrive at a draft version of the GDIT. During the meetings, Delphi item ratings and categories of item issues identified in expert comments from the Delphi questionnaire were discussed in detail for each item, in relation to the recommendations in the Banff consensus (Walker et al., 2006). A PowerPoint presentation was used as a tool to summarize items, problematic issues and proposed solutions (see Figure S1). At each consensus meeting, the discussion involved how to resolve the item issues identified in the expert comments, which frequently concerned item phrasing or formulation of response categories, as well as whether to include the item in the draft version of the GDIT. The discussion ended in a consensus‐based outcome for each item. Thereafter, a draft version of the GDIT was formulated.

### 2.3 User experience and pilot testing

To evaluate user experience and face validity, the draft GDIT English version was translated into Swedish using a back‐translation procedure. The Swedish version was then presented to participants with self‐experienced PG, recruited from self‐help groups (*n* = 12), using a "think aloud" procedure (Boren & Ramey, 2000; Ericsson & Simon, 1980). The interviews were conducted by authors OM and VM at the local Association for Gambling Addiction in Stockholm and the Center for Dependency Disorders in Falun. In order to assess feasibility and face validity of the GDIT draft version, it was then administered to a small sample (*n* = 8) of treatment‐seeking gamblers at the Stockholm Center for Dependency Disorders. This procedure constituted a purely qualitative test of the draft version and as such the sample sizes were deemed sufficient when participant comments were saturated.

### 2.4 Data analysis

Frequencies, means, standard deviations as well as “critical for inclusion” percentages reflecting item ratings of 7–9, in Delphi rounds 1 and 2 were calculated. All quantitative analyses were done in R Studio version 1.1.456 (R Core Team, 2018). Qualitative analysis of the Delphi expert comments of issues in the proposed items was conducted by author OM, using a simple review and categorization procedure. Participant responses in the “think aloud” interviews as well as data from the psychometric pilot were reviewed by author OM with the aim of identifying and addressing remaining item issues, and subsequently discussed with author AHB in order to reach consensus decisions for each remaining issue.

## 3 RESULTS

### 3.1 Quantitative Delphi analysis

Of the 170 invited stakeholders, 61 stakeholders consented and completed the first Delphi round, and 30 of these completed the second Delphi round (49% completion rate). Stakeholders included gambling researchers, clinicians and trainers from 10 countries (31% women). Table 1 shows participant characteristics from the first and second rounds.

**TABLE 1:** Participants in Delphi rounds, consensus meetings and evaluation of user experience

	Delphi		Consensus meetings		User experience	
	Round 1 (*n* = 61)	Round 2 (*n* = 30)	Banff (*n* = 10)	Stockholm (*n* = 4/3)^a^	“Think aloud” (*n* = 12)	Pilot (*n* = 8)
Country						
Australia	10 (16%)	5 (17%)	‐	‐	‐	‐
Canada	11 (18%)	3 (10%)	5 (50%)	1/0^a^	‐	‐
England	1 (2%)	1 (3%)	1 (10%)	‐	‐	‐
France	1 (2%)	1 (3%)	‐	‐	‐	‐
Germany	1 (2%)	0 (0%)	‐	‐	‐	‐
New Zealand	2 (3%)	1 (3%)	‐	‐	‐	‐
Norway	2 (3%)	0 (0%)	‐	‐	‐	‐
Spain	1 (2%)	1 (3%)	‐	‐	‐	‐
Sweden	26 (43%)	16 (53%)	3 (30%)	3	12	8
USA	6 (10%)	2 (7%)	1 (10%)	‐	‐	‐
Gender						
Men	42 (69%)	20 (67%)	5 (50%)	3 (2)^a^	^c^	7
Women	19 (31%)	10 (33%)	5 (50%)	1	^c^	1
Professional role^b^						
Researchers	58 (95%)	27 (90%)	10 (100%)	^c^	‐	‐
Clinicians	17 (28%)	12 (40%)		^c^	‐	‐
Trainers	12 (20%)	7 (23%)		^c^	‐	‐
Experience of working with gambling						
<5	14 (23%)	9 (30%)	^c^	^c^	‐	‐
5–9	11 (18%)	5 (17%)	^c^	^c^	‐	‐
10–19	20 (33%)	7 (23%)	^c^	^c^	‐	‐
≥20	16 (26%)	9 (30%)	^c^	^c^	‐	‐

^a^ In the second consensus meeting, four researchers participated, one from Canada and three from Sweden; in the third consensus meeting, three researchers participated, all from Sweden.

^b^ Expert stakeholders could indicate multiple roles, so the total exceeds 100%.

^c^ This data was not collected during this phase of the study.

The consensus process led to selection of 10 items, deriving from six different prior instruments (PPGM 10a, SOGS 4, PPGM 8, MAGS 25, PPGM 1b, CSPG 1, CPGI 8, NODS 14, CPGI 10, and “Gambling types”) that fulfilled the criteria for consensus regarding importance of inclusion in the GDIT (see Table 2). These 10 items targeted the following constructs, listed in order of rating level, from highest to lowest: Loss of control, Chasing losses, Jeopardized opportunities, Financial problems, Frequency of gambling behavior, Tolerance, Relationship problems, Borrowed/Sold articles of value and Gambling types. Most items that fulfilled the criteria for consensus regarding importance of inclusion in the GDIT were in the domains of dependence symptoms (*n* = 4) and negative consequences (*n* = 4). None of the items targeting the constructs of Preoccupation or Expenditures were rated highly enough in terms of importance to be included in the GDIT. In general, all items targeting monetary constructs (e.g., losses, spending, income or net expenditures), were rated low in both Delphi questionnaire rounds 1 and 2 (mean < 6 on a scale from 1 to 9). Comments concerning the low ratings for monetary constructs suggested that such constructs are difficult to measure since they are complicated constructs liable to misinterpretation in terms of the time frame (e.g., gambling session length), spending versus winning/losing, impose a high cognitive load for the respondent due to this complexity, are vulnerable due to lack of verifiability regarding monetary expenditures and, finally, are plagued by recall bias.

**TABLE 2:** Expert ratings and comments on item‐related problematic issues in Delphi rounds 1 and 2

Expert ratings Delphi									
Delphi content				Round 1 (*n* = 61)		Round 2 (*n* = 30)		Item‐related issues	
Domain	Construct	Nr	Item^a^	M (Sd)	CFI^b^	M (Sd)	CFI^b^	Expert comments (selective)	Categories
Dependence symptoms^c^	Loss of control	11	How often have you made attempts to cut down, control or stop your gambling, the past 12 months? [PPGM10a]	7.52 (1.79)	80	8.33 (0.76)	97	Very relevant. Suggest possibly a follow‐up question to ask how successful these attempts were. Stakeholder 24	Phrasing
Dependence symptoms^c^	Chasing losses	8	How often have you gambled to win back money you lost, the past 12 months? [SOGS4]	7.8 (1.64)	87	7.97 (1.69)	87	The final phrase should read, “in the past 12 months”. Does the 12‐month time frame make follow up over shorter periods difficult? Stakeholder 50	Phrasing, time frame
Dependence symptoms^c^	Loss of control	10	How often have you gambled longer, with more money or more frequently than you intended to, the past 12 months? [PPGM8]	7.16 (2.08)	79	7.87 (1.11)	87	It's a double‐barrelled question, but tapping into the right area. The sense of increase is important to capture. Stakeholder 54	Compound
Negative consequences	Jeopardized opportunities	15	Have you jeopardized or lost a significant relationship, job, educational or career opportunity because of your gambling? [MAGS25]	7.25 (1.96)	77	7.8 (1)	87	I do not think relationship and employment consequences should be mixed up. They are very different impacts, with different levels of frequency (…). Stakeholder 8	Compound
Negative consequences	Financial concerns	21	Has your gambling caused significant financial concerns for you or someone close to you? [PPGM1b]	7.07 (1.78)	74	7.57 (1.48)	90	Relevant, two constructs in one item. Could be rephrased. Stakeholder 38	Compound, phrasing
Gambling consumption behaviors^d^	Gambling behavior: Frequencies	1	How often do you gamble now? [CSPG1]	7.44 (1.91)	80	7.47 (1.31)	80	It is unclear what "now" means. In the past year? In the past month? Stakeholder 58	Time frame
Expenditures and gambling types	Gambling types	30	Please report which types of gambling that have been problematic to you. Do not report gambling types that have not led to negative consequences. If several gambling types have been problematic to you, report all of these^e^	6.79 (2.02)	61	7.27 (1.74)	80	This is an important question but when I read the comments from other I suggest dropping "problematic" and instead asking about harm and negative consequences. “Problematic” might lead the thought to problems with game play, such as selecting the winning horses in horse racing. Stakeholder 11	Phrasing
Dependence symptoms^c^	Tolerance	9	How often have you needed to gamble with larger amounts of money to get the same feeling of excitement, the past 12 months? [CPGI8]	7.07 (1.7)	70	7.23 (1.59)	77	This is really a yes or no question but its asking for an answer in terms of frequency (…). Stakeholder 31	Response categories
Negative consequences	Relationship problems	22	Has your gambling caused serious or repeated problems in your relationships with any of your family members or friends? [NODS14]	7.07 (1.7)	69	7.23 (1.96)	77	Double‐barreled. Serious or repeated? Maybe one, maybe both? (…). Stakeholder 22	Compound, phrasing
Negative consequences	Borrowed/sold	20	Have you borrowed money or sold anything to get money to gamble? [CPGI10]	6.97 (1.63)	67	7.17 (1.49)	77	Perhaps I would have used more gradients in the scale. Stakeholder 36	Response categories
Dependence symptoms^c^	Escape	14	How often have you gambled as a way of escaping from problems or relieving feelings of helplessness, guilt, anxiety or depression, the past 12 months? [MAGS21]	6.79 (2.04)	64	6.93 (1.68)	67	I'd prefer the simpler "as a way of escaping problems" to avoid the double‐barreled issues with this question. Stakeholder 21	Compound, phrasing
Dependence symptoms^c^	Lies	13	How often have you lied to family members, friends, or others about how much you gamble or how much money you lost on gambling, the past 12 months? [NODS11]	7.3 (1.44)	79	6.9 (2.2)	80	Greatly depends on the kinds of relationships the person has? Someone with a spouse versus someone without for example. Stakeholder 53	Phrasing
Negative consequences	Suicide	19	Have you seriously thought about or attempted suicide as a result of your gambling? [CPGI32/CPGI33]	6.34 (2.11)	52	6.63 (2.03)	57	The GDIT would presumably be used in conjunction with other self‐rating scales and a suicidality question feels out of place here. Stakeholder 14	Lack of relevance/applicability
Negative consequences	Worries	17	Has a relative or a friend, a health care or social worker, or anyone else, expressed worries about your gambling or told you that you should stop gambling? [AUDIT10/DUDIT11]	6.64 (1.64)	61	6.6 (1.38)	57	This item is tricky. Gamblers try to hide their habits and do not listen to warnings (…). Stakeholder 42	Lack of relevance/applicability
Negative consequences	Health problems	23	Has your gambling caused you any health problems, including stress or anxiety? [CPGI16]	6.49 (2.15)	61	6.57 (2.01)	60	Ordinary people wouldn't describe stress and anxiety as "health problems". Could you say "including mental problems such as stress or anxiety"? What other kinds of health problems are intended to be included? (…). Stakeholder 35	Phrasing
Dependence symptoms^c^	Abstinence	12	How often when you were not gambling the past 12 months, did you experience irritability, restlessness or strong cravings to gamble? [PPGM13]	6.77 (1.78)	66	6.47 (2.05)	67	The question looks double‐barrelled. It captures two different things. But it's a highly relevant area. Cravings don't always equate to irritability. Stakeholder 54	Compound
Negative consequences	Mental health problems	18	Has your gambling caused significant guilt, anxiety, or depression symptoms for you or someone close to you? [PPGM2]	6.44 (1.87)	54	6.43 (1.5)	53	I think it would be better to separate the individual harm from the harm caused third party. Stakeholder 13	Compound
Dependence symptoms^c^	Preoccupation	5	How often have you spent at least one hour thinking about your gambling experiences, or planning future gambling ventures or bets, the past 12 months? [NODS1]	6.05 (2.03)	51	6.13 (1.28)	47	Corresponding diagnostic criteria has high discriminative function, but not sure about one‐hour threshold. Is it empirically derived? Stakeholder 14	Time frame
Gambling consumption behavior^d^	Gambling behavior: time	2	How much time do you now spend gambling on a typical day? [CSPG2]	6.75 (1.87)	61	6.03 (1.85)	40	Recommend providing categories above the 3 h category as many problem gamblers will be doing in excess of this figure. Suggest a 6+ hrs category as the maximum. Stakeholder 24	Response categories
Dependence symptoms^c^	Preoccupation	6	How often have you been preoccupied with gambling, the past 12 months? [PPGM12/BPGS 1]	5.67 (2.13)	43	5.97 (1.85)	40	I think this item should be more specific about what constitutes "preoccupation" (e.g., "thinking about gambling when you are doing other activities"). Stakeholder 31	Phrasing
Expenditures and gambling types	Income	29	What is your monthly income?^f^	5.82 (2.41)	51	5.77 (2.16)	43	Important for determining gambling expenditure relative to income. Respondents could answer based on before tax or after tax income, so specify which you want them to provide. Stakeholder 30	Phrasing
Dependence symptoms^c^	Preoccupation	7	How often have thoughts of gambling been constantly in your mind, the past 12 months? [VGS8]	5.54 (2.43)	43	5.73 (2.2)	40	I would have used; repeatedly, several times a day (or equivalent) as the most frequent statement. Stakeholder 36	Response categories
Gambling consumption behaviors^d^	Gambling behavior: time	4	Approximately how much total time do you spend gambling or on gambling related activities, during one week? [GSAS6]	5.57 (1.67)	33	5.5 (1.74)	40	These are two separate questions ‐ double barrelled. Need to define gambling‐related activities and ask separately. Stakeholder 28	Compound
Negative consequences	Harms	16	Have you or anyone else been harmed (mentally or physically) because of your gambling? [AUDIT9/DUDIT10]	6.28 (2.26)	56	5.47 (2.1)	30	Might work better as two questions: Perceived harm to self and perceived harm to others such as family members. Stakeholder 5	Compound
Gambling consumption behaviors^d^	Gambling behavior: time	3	How often do you now spend more than 2 h gambling, in a single session? [CSPG3]	5.69 (1.98)	34	5.37 (1.83)	30	My main concern with this item is the selection of 2 h as my understanding is that this cut‐off was selected arbitrarily (…). Stakeholder 56	Time frame
Expenditures and gambling types	Gambling spendings/losses	25	How much money, not including winnings, have you spent on gambling the last month? [CPGI4]	5.38 (2.6)	46	5.37 (2.24)	30	This question has always been confusing to participants, as certain forms of gambling (e.g., EGMs) it is very difficult to separate out winnings from just outlay. Stakeholder 31	Lack of relevance/applicability
Expenditures and gambling types	Gambling spendings/losses	27	Approximately how much money have you spent (lost) gambling in the past month [GQPN5]?	5.25 (2.42)	33	5.13 (2.3)	30	This item will likely capture biased responses if the participant has won any amount of money in the past month. Stakeholder 4	Lack of relevance/applicability
Expenditures and gambling types	Net expenditure	26	In the past 12 months, how much money do you estimate you spent on gambling in a typical month? Spent means how much you are ahead or behind or your net win or loss (if you have a net win, put a + sign in front of the number). [GPI1e]	4.66 (2.5)	31	4.6 (1.81)	13	Specific instruction, but somewhat complicated. Stakeholder 33	Phrasing
Expenditures and gambling types	Gambling spendings	28	How much money have you spent on gambling the last week? [PGBS1]	4.64 (2.49)	25	4.13 (2.27)	17	Putting (lost) in parentheses after this question does improve it. The wording of this question is likely to yield the most unreliable results. Stakeholder 24	Lack of relevance/applicability, Phrasing
Expenditures and gambling types	Currency	24	What is your currency (eg, SEK, NOK, Euro, AUD, etc.)?^f^	4.33 (2.53)	21	3.7 (1.74)	0	This problem could be solved with currency specified for the translated language versions, respectively, instead. Stakeholder 33	Lack of relevance/Applicability

Abbreviations: AUDIT, the Alcohol Use Disorders Identification Test; BPGS, the Brief Problem Gambling Screen; CFI, critical for inclusion; CPGI, the Canadian Problem Gambling Index; CSPG, the Consumption Screen for Problem Gambling; DUDIT, the Drug Use Disorders Identification Test; GDIT, Gambling Disorder Identification Test; GPI, the Gambling Participation Instrument; GQPN, the Gambling Quantity and Perceived Norms Scale; G‐SAS, the Gambling Symptom Assessment Scale; MAGS, the Massachusetts Gambling Screen; NODS, the NORC Diagnostic Screen for Gambling Problems; PGBS, the Pathological Gambling Behavioural Self‐Report Scale; PPGM, the Problem and Pathological Gambling Measure; SOGS, the South Oaks Gambling Screen; VGS, the Victorian Gambling Screen.

^a^ All items were rephrased to fit the GDIT format.

^b^ CFI: percentage of Delphi participants rating the item as 7, 8 or 9.

^c^ Domain was presented as Gambling consumption behaviors in the Delphi and later revised as Gambling behavior in the GDIT draft version.

^d^ Domain was presented as Dependence symptoms in the Delphi and later revised as Gambling symptoms in the GDIT draft version.

^e^ item constructed based upon the Gambling Participation Instrument (Williams et al., 2017).

^f^ Item constructed, AUDIT (Saunders et al., 1993), BPGS (Volberg & Williams, 2011), CPGI (Ferris & Wynne, 2001), CSPG (Rockloff, 2012), DUDIT (Berman et al., 2005), GPI (Williams et al., 2017), GQPN (Neighbors, Lostutter, Larimer, & Takushi, 2002), G‐SAS (Suck Won Kim, Grant, Potenza, Blanco, & Hollander, 2009), MAGS (Shaffer, LaBrie, Scanlan, & Cummings, 1994), NODS (D. C. Hodgins, 2004), PGBS (Hollander, Pallanti, Allen, Sood, & Rossi, 2005), PPGM (Williams & Volberg, 2013), SOGS (Lesieur & Blume, 1987), VGS (Tolchard & Battersby, 2010).

### 3.2 Qualitative Delphi analysis

A range of potential problematic issues in relation to the items rated in the Delphi questionnaire was identified in the expert comments, yielding six categories: Time frame, Response categories, Compound formulation (referring to double‐ or triple‐barreled items), Phrasing, Lack of relevance/applicability, and Other/miscellaneous (see Table 2). Typically, comments on items in the domains of Dependence symptoms and Negative consequences were categorized in the Phrasing and Compound formulation categories, while comments on items in the Gambling consumption behaviors domain were categorized in the Time‐frame and Response categories. Comments on items in the Expenditures and gambling types domain mainly belonged to the category of Lack of relevance/applicability.

### 3.3 Item selection

The 10 items that fulfilled consensus criteria regarding importance of inclusion in the Delphi were reviewed by author OM in relation to the recommendations in the Banff consensus (Walker et al., 2006). Several recommended constructs were lacking, for example, Preoccupation, Expenditures, and Health problems due to gambling, leading to construct under‐representation in relation to the Banff recommendations. Therefore, 11 additional items below the Delphi consensus threshold (NODS 11, PPGM 12/BPGS 1, MAGS 21, CSPG2, PPGM2, "Income", CPG I4, GPI 1e, GQPN 5, PGBS 1, and CPGI 32/CPGI 33; see Table 3 below) were added to be considered for inclusion in GDIT in the three consensus meetings. Modified response categories, analogous to the AUDIT (Saunders et al., 1993) and the DUDIT (Berman et al., 2005) format, were also proposed for all the selected items within the GDIT domains Gambling consumption behavior, Dependence symptoms and Negative consequences.

**TABLE 3:** Item selection flow in the GDIT Delphi process

Delphi	Consensus meetings		Pilot testing
30 items	Banff 6 items	Stockholm 14 items + 3 appendix items	GDIT draft version 14 items (constructs) +5 appendix items
**Gambling consumption behaviors** CSPG 1, CSPG 2, CSPG 3, G‐SAS 6	‐	CSPG 1, CSPG 2	**Gambling behavior** CSPG 1 (frequency of gambling behavior), CSPG 2 (time for gambling behavior), PPGM 12/BPGS 1 (time for preoccupation)
**Dependence symptoms** CPGI 8, MAGS 21, NODS 1, PPGM 10a, NODS 11, PPGM 8, PPGM 12/BPGS 1, PPGM 13, SOGS 4, VGS 8	PPGM 10a, PPGM 8, SOGS 4	CPGI 8, MAGS 21, PPGM 10a, NODS 11, PPGM 8, PPGM 12/BPGS 1, SOGS 4	**Gambling symptoms** CPGI 8 (tolerance), CPGI 10 (borrowed/sold), MAGS 21 (escape), PPGM 10a (loss of control), NODS 11 (lies), PPGM 8 (loss of control), SOGS 4 (chasing losses)
**Negative consequences** AUDIT 9/DUDIT 10^a^, AUDIT 11/DUDIT 11^a^, CPGI 10, CPGI 32, MAGS 25, NODS 14, PPGM 1, PPGM 2	MAGS 25, NODS 14, PPGM 1	CPGI 10, MAGS 25, NODS 14, PPGM 1, PPGM 2	**Negative consequences** MAGS 25 (jeopardized opportunities), NODS 14 (relationship problems), PPGM 1 (financial problems), PPGM 2 (mental health problems)
**Expenditure and gambling types** CPGI 13, gambling types and list^b^, GPI 1, GQPN 5, monthly income^c^, national currency^c^, PGBS 1	‐	Gambling types and list^b^, GQPN 5, monthly income^c^	**Appendix: Expenditures and gambling types** Bets^d^, ^e^, gambling types and list^b^, ^d^, losses^d^, ^e^, monthly income^c^, ^d^, wins^d^, ^e^

Abbreviations: AUDIT, the Alcohol Use Disorders Identification Test; BPGS, the Brief Problem Gambling Screen; CPGI, the Canadian Problem Gambling Index; CSPG, the Consumption Screen for Problem Gambling; DUDIT, the Drug Use Disorders Identification Test; GDIT, Gambling Disorder Identification Test; GPI, the Gambling Participation Instrument; GQPN, the Gambling Quantity and Perceived Norms Scale; G‐SAS, the Gambling Symptom Assessment Scale; MAGS, the Massachusetts Gambling Screen; NODS, the NORC Diagnostic Screen for Gambling Problems; PGBS, the Pathological Gambling Behavioural Self‐Report Scale; PPGM, the Problem and Pathological Gambling Measure; SOGS, the South Oaks Gambling Screen; VGS, the Victorian Gambling Screen.

^a^ Item constructed to fit gambling based upon the AUDIT (Saunders et al., 1993) and the DUDIT (Berman et al., 2005).

^b^ Item constructed based upon the Gambling Participation Instrument (Williams et al., 2017).

^c^ Item constructed.

^d^ Item added to address the recommendations in the Banff consensus (Walker et al., 2006).

^e^ Item constructed based upon the TimeLine Followback for Gambling (D. Hodgins, 2014), AUDIT (Saunders et al., 1993), BPGS (Volberg & Williams, 2011), CPGI (Ferris & Wynne, 2001), CSPG (Rockloff, 2012), DUDIT (Berman et al., 2005), GDIT (Molander et al., 2019), GPI (Williams et al., 2017), GQPN (Neighbors et al., 2002), G‐SAS (Suck et al., 2009), MAGS (Shaffer et al., 1994), NODS (D. C. Hodgins, 2004), PGBS (Hollander et al., 2005), PPGM (Williams & Volberg, 2013), SOGS (Lesieur & Blume, 1987), VGS (Tolchard & Battersby, 2010).

### 3.4 Consensus meetings

Three consensus meetings were held. The first meeting, held in Banff, included 10 gambling researchers from Canada, England, Sweden and the USA. The outcome of this meeting was the inclusion of six items fulfilling Delphi criteria in the draft GDIT version (PPGM 10a, SOGS 4, PPGM 8, MAGS 25, PPGM 1b, and NODS 14). Two changes in item phrasing were implemented: PPGM 8 was rephrased to avoid compound formulation and PPGM 1b was moved from the GDIT Dependence symptoms domain to the Negative consequences domain. Also, a discussion was held concerning whether to expand response categories in the Gambling consumption behaviors domain, as gambling may occur more frequently than alcohol or drug use. The second meeting, held in Stockholm, included four gambling researchers from Sweden and Canada. The outcome of this meeting was the inclusion of four remaining items which fulfilled the Delphi criteria in the draft GDIT version (CSPG 1, CPGI 8, “Gambling types,” and CPGI 10). Two items that were rated below the Delphi consensus threshold (NODS 1 and PPGM 12) were reviewed and included, based on their alignment with the Banff recommendations (Walker et al, 2006). In addition, CPGI 8 was moved from the GDIT Dependence symptoms domain to the Negative consequences domain, and CPGI 10 was moved from the Negative consequences domain to the Dependence symptoms domain. The instructions for the “Gambling types” item were rephrased and the “Gambling list” was reviewed and revised to improve categories and examples of gambling types. The third and final consensus meeting included three gambling researchers from Sweden, who reviewed the remaining items that were rated below the Delphi consensus threshold. Five of these (MAGS 21, CSPG 2, PPGM 2, “Income,” and GQPN 5) were included in the draft version of the GDIT. The constructed “Income” item was rephrased as “income after tax,” including salary and welfare or other subsidies, and GQPN 5 was rephrased to only assess losses rather than spending and losses, to clarify the question and reduce confusion. At each meeting, included items were rephrased and clarified to match the GDIT format. Following the consensus meetings, the GDIT draft version in English was translated into Swedish using a back‐translation procedure (Kuliś, Whittaker, Greimel, Bottomley, & Koller, 2017).

### 3.5 Preliminary testing and final draft version

Participants with personal experience of PG (*n* = 12) were recruited from gambling self‐help groups, and gave feedback on each item in the GDIT draft version according to a “think aloud” procedure (Ericsson & Simon, 1980). Overall, the participants expressed that the items in the GDIT draft version were comprehensible and important from PG and GD perspectives. The participants also suggested that response alternatives should be added in the Gambling behavior domain to include gambling every day, and discussed whether gamblers could reliably estimate and report gambling losses in the expenditures and gambling types appendix. See Table 4 for examples of participant responses.

**TABLE 4:** Examples of participant comments in “think aloud” interviews

GDIT draft version			
Domain	Item	Response categories	“Think aloud” comments (selective)
Gambling behavior	1. How often do you gamble?	Never, monthly or less, 2–4 times a month, 2–3 times a week, 4 or more times a week	Well, I gambled continuously. 4 times a week (response category) maybe to little? Maybe add hours? Many gamblers are gambling everyday, for a very long time (Participant 11)
Gambling behavior	2. How much time do you spend gambling on a typical day?	No time, Less than an hour, 1–2 h, 3–4 h, 5–6 h, 7 or more hours	Same here actually (as item 1). I gambled around the clock as a poker player. (Add) “around the clock” or “8, 10 or 12 h” as response alternative (Participant 5)
Gambling symptoms	9. How often have you gambled as a way of escaping problems or relieving negative feelings, in the past 12 months?	Never, Less than monthly, monthly, weekly, daily or almost daily	Daily! Here everything is perfect, this whole question. Also the response alternatives (Participant 8)
Negative consequences	11. Have you or anyone close to you experienced financial problems due to your gambling?	No, yes but not in the past year, yes in the past year	My though here is, yes I will have problems the following 7–8 years… it follows you quite long. Just ask “have your gambling led to economic problems.” (Participant 4)
Expenditures and gambling types	What was your income after tax last month (including salary and grants)? How much money did you lose on gambling last month?	_$, _$	Income is possible to report. If you're not counting income from gambling. Amounts of money lost on gambling are hard to keep track on, until you stop (gambling). Also (gamblers) gladly only report wins. It could be good to include (item) anyhow as a form of consequence, “loose the blinders.” Also, is it important how you feel? (Participant 10)
Expenditures and gambling types	What was your income after tax last month (including salary and grants)? How much money did you lose on gambling last month?	_$, _$	Oh. As I said earlier, an addicted gambler will not be able to report this honestly. You don't see the losses either. You don't count the small amounts, only the big ones. So it's hard. Few can keep track of their gambling. Also it could be a trigger (amounts—gambling). But it's good to make (losses) visible. It's common to beautify and deny what you lost (Participant 2)

The GDIT draft version was then administered to a subsample (*n* = 8) of treatment‐seeking gamblers, in a pilot test. The participant responses were reviewed and remaining issues with the expenditure items in the GDIT appendix were identified. Following this preliminary testing, involving evaluation of user experience and pilot testing, some final adjustments were made in the GDIT draft version based on consensus decisions by authors OM and AHB. First, to address the issue that gambling behavior might occur more frequently than use of alcohol or drugs, the response categories for the items in the Gambling behavior domain were revised. For item 1, two response alternatives (“Daily,” “Several times a day”) were added to further specify frequency of gambling behavior. For items 2 (time for gambling behavior) and 3 (time for preoccupation), one response category (“10–24 h”) was added in order to include gambling behavior that occurs during an entire 24‐h period. Second, in an effort to fully comply with the specifications of the Banff consensus regarding expenditures (Walker et al., 2006), the initial draft GDIT appendix item measuring past month losses was replaced with three items (past month “Bets”, “Wins” and “Losses”), constructed based on the TimeLine FollowBack method adapted for gambling (D. Hodgins, 2014; D. C. Hodgins & Makarchuk, 2003; Weinstock, Whelan, & Meyers, 2004).

The final GDIT draft version consisted of two pages, printed front and back in the paper version. Page 1 consisted of 14 items in three domains: gambling behavior, gambling symptoms and negative consequences, and used multiple choice frequency‐based response alternatives similar to the AUDIT (Saunders et al., 1993) and the DUDIT (Berman et al., 2005). Page 2 consisted of an appendix with four items measuring past month expenditures and one item concerning gambling types, showing a detailed list defining examples and categories of gambling types.

## 4 DISCUSSION

This article describes an iterative collaborative consensus process for specific item selection and modification in the development of a new gambling measure. A specific set of items with the highest priority was identified and included in a testable draft version of the GDIT. Overall, the study established preliminary construct and face validity for the GDIT, with item domains that align with the constructs in the Banff consensus recommendations, as well as the AUDIT and DUDIT domains of consumption, symptoms and negative consequences.

Two major item‐related issues were identified and addressed. First, it became evident that many Delphi items, gathered from existing gambling measures, were phrased using double or triple compound phrasing. Some possible explanations for this could be that items were originally phrased in an effort to clarify their construct using examples, or that they emanated from the diagnostic criteria formulated in a compound manner, for example A8 “Has jeopardized or lost a significant relationship, job, or educational or career opportunity because of gambling” (American Psychiatric Association, 2013). However, while assessing items, many Delphi stakeholders emphasized that double or triple compound formulation of items can be problematic. Several participants in the “think aloud” interviews also remarked on this issue, stating for example that it was confusing to know which of the statements or examples to answer. We addressed compound formulation issues, when applicable, by rephrasing the items so that they targeted a single construct of primary interest (e.g., gambling‐related negative consequences for relationships), in an effort to strengthen the construct validity of the GDIT draft version.

Second, items targeting expenditures were frequently identified as problematic by participants throughout all phases in the Delphi process. The Banff consensus (Walker et al., 2006) states that net expenditure each month should be reported as cash in minus cash out. The Banff consensus also states that financial losses should refer to net losses: “the actual amount of money the gambler brings to a session (which includes cash or cash equivalents such as cheques or money orders plus subsequent withdrawals or borrowings) less the actual amount remaining at the conclusion of the session.” However, all items targeting expenditures were rated low among the expert stakeholders in the Delphi, with some Delphi stakeholders even arguing against including expenditures in the GDIT. Gambling expenditures have been investigated in several studies (e.g., Blaszczynski, Ladouceur, Goulet, & Savard, 2006; Williams, Volberg, Stevens, Williams, & Arthur, 2017; Wood & Williams, 2007) showing a lack of correspondence between self‐reported gambling expenditures and actual revenue. Measuring expenditures in gambling research is complicated, as gamblers may not be able to remember or estimate their gambling expenditures accurately. Other possible sources of self‐report biases among gamblers could be positive memory bias (not thinking about or reporting losses) (Boffo et al., 2018), or not fully understanding instructions on how to estimate theoretical constructs such as net expenditure or net losses. These issues, related to reporting gambling expenditures, were emphasized by several participants in the “think aloud” interviews and by many Delphi stakeholders. Somewhat surprisingly, items with more detailed instructions, such as the GPI 1e (Williams et al., 2017), were rated as less important than expenditure items using vague or compound formulation by the expert Delphi stakeholders, who commented that items with highly detailed instructions seemed too complicated. To address these issues and in an effort to comply with the Banff consensus (Walker et al., 2006), a final decision was made to replace all expenditure items in the draft version of the GDIT appendix with three new self‐report items assessing monetary sums (past month wagers on gambling, past month winnings on gambling, and past month gambling losses), as a condensed form of the Timeline FollowBack (TLFB; Sobell & Sobell, 1996). Briefly, the TLFB is a retrospective interview method originally developed to assess alcohol use, using calendar and memory aids, which was later adapted and applied to other addictive behaviors such as gambling (D. C. Hodgins & Makarchuk, 2003; D. Hodgins, 2014; Weinstock et al, 2004). In a psychometric evaluation among frequent gamblers, Weinstock et al, (2004), found that the TLFB for gambling demonstrated adequate to excellent test‐retest reliability (*r* = 0.75–0.96), and correlated positively with daily self‐monitoring reports, as well as other gambling screening instruments. In addition, we included one monetary item assessing past month income, to be able to compare gambling expenditure in relation to income, as suggested in the Banff consensus (Walker et al., 2006).

This study was characterized by numerous strengths. First, initial item selection was based upon a comprehensive analysis of existing self‐report instruments for measuring PG and GD. This analysis included inter‐rater reliability calculations regarding content of specific items (Molander et al., 2019), referencing of previous psychometric findings (Chamberlain et al., 2017; Stinchfield et al., 2016; Volberg & Williams, 2011) and previous consensus‐based frameworks among gambling researchers (Walker et al., 2006), as well as taking the revised DSM‐5 criteria for GD into account (American Psychiatric Association, 2013). Second, an international group of experts from a total of ten countries participated in the Delphi survey, in many cases giving detailed, specific feedback on each individual item. Third, transparent procedures were applied for arriving at consensus‐based decisions. Fourthly, we used think‐aloud interviews to gather feedback from participants with experience of PG, in an effort to increase the face validity of the GDIT draft version, which led to extension of the response alternatives on the gambling behavioral frequency items to include multiple sessions during a 24‐h period, as well as considering revision of the expenditure items to follow TLFB procedures. Further, pilot psychometric testing from participants with both PG and GD convinced us to revise the expenditure items, as the initial responses were very difficult to interpret. Fifth, structured consensus procedures were used to resolve item‐related issues that were identified throughout the phases in the study, as well as to address the recommendations in the Banff consensus (Walker et al., 2006). An additional strength concerns the research strategy from a wider perspective. As noted above, the gambling research field encompasses a large number of diverse measures and outcomes (Molander et al., 2019; Otto et al., 2020; Pallesen et al., 2005; Pickering et al., 2017), making it difficult to synthesize research findings, for example in systematic reviews and meta‐analyses of trial outcomes. This problem has also been identified in the area of hazardous and harmful alcohol consumption, and is being addressed by an initiative to establish a minimum set of core outcomes for wide use in treatment outcome studies (Shorter et al., 2019). The problem of measure diversity not only hinders comparability, it also contributes to researchers spending valuable time and resources collecting data and analyzing results that may not make as great a contribution as desired. By joining forces, the research field can avoid "reinventing the wheel" and combine forces to advance the gambling studies field. In sum, the development of the GDIT has the potential to resolve some of the field's current challenges related to measurement. Some limitations also characterized this study. First, it was not possible to reach a broad consensus‐based conclusion on how to measure gambling expenditures on a specific item level, reflecting the complexity of this issue. Secondly, although a fairly large number of expert stakeholders participated in the Delphi, only about half completed the second round; this could conceivably have yielded a biased sample but the participant characteristics from rounds 1 and 2 were approximately equivalent in terms of country, gender, professional role and years of experience working with gambling issues. Due to limited time before the planned in‐person consensus meeting in Banff, the available period for stakeholders to complete the Delphi rounds was short. Thirdly, it would have been preferable to include all gambling researchers from the consensus meeting in Banff in all consensus‐based decisions regarding the GDIT, but this was not possible due to practical and time‐related challenges. Finally, the use of a predetermined rating scale (see Guyatt et al, 2011) for item inclusion in the Delphi, may have made it more difficult to include items of content‐based value in relation to the Banff consensus (Walker et al., 2006). Had we chosen a more data driven approach for establishing a tailored rating scale, the results of the Delphi process might have been more aligned with the recommendations of Walker et al. (2006).

Future psychometric studies on the GDIT instrument will evaluate the validity and reliability of the GDIT against the DSM‐5 criteria for GD, a study that is ongoing as step 4 in the development process and expected to generate instructions for scoring and application in clinical and public health settings. Additional studies of interest would include evaluation of expenditure items against objective monetary measures such as bank or gambling accounts, examining sensitivity to change following interventions, as well as translation and cultural adaptation of the GDIT to other languages.

## CONFLICT OF INTEREST

The authors identify no conflicts of interest. Author Anne H. Berman is a board member of the independent research council funded by the state‐owned gambling company Svenska Spel AB. The council funds gambling‐related studies but the company Svenska Spel AB has no influence whatsoever on decisions to grant funds to researchers. All authors except Olof Molander and Rachel Volberg are recipients of prior grants from this research council for separate independent studies, unrelated to the work reported in this article.

## ETHICAL APPROVAL

This study was approved by the Regional Ethics Board of Stockholm, Sweden (ref. no. 2017/1479‐31). Approval was granted for all stages of the study. All participants provided informed consent for participation and publication.

## AUTHOR CONTRIBUTIONS

Olof Molander and Anne H. Berman conceived the study. Olof Molander and Anne H. Berman prepared the Delphi survey, in collaboration with the other authors. Rachel Volberg provided expert guidance on the methodology as an experienced gambling researcher and developer of gambling measures. Olof Molander, Anne H. Berman and Rachel Volberg hosted the Banff consensus meeting and Olof Molander and Anne H. Berman hosted the Stockholm consensus meetings. Olof Molander and Viktor Månsson conducted the “think aloud” interviews. Olof Molander analyzed the results. Olof Molander wrote the first manuscript draft, and Anne H. Berman and Viktor Månsson revised the second draft. All authors edited and contributed to subsequent manuscript drafts.

## Supporting information

Supplementary Material

## References

[mpr1865-bib-0001] American Psychiatric Association . (2013). Diagnostic and statistical manual of mental disorders (5th ed.). Arlington, VA: American Psychiatric Association.

[mpr1865-bib-0002] Berman, A. H. , Bergman, H. , Palmstierna, T. , & Schlyter, F. (2005). Evaluation of the drug use disorders identification test (DUDIT) in criminal justice and detoxification settings and in a Swedish population sample. European Addiction Research, 11(1), 22–31. 10.1159/000081413 15608468

[mpr1865-bib-0003] Blaszczynski, A. , Ladouceur, R. , Goulet, A. , & Savard, C. (2006). ‘How much do you spend gambling?’: Ambiguities in questionnaire items assessing expenditure. International Gambling Studies, 6(2), 123–128. 10.1080/14459790600927738

[mpr1865-bib-0004] Blaszczynski, A. , & Nower, L. (2002). A pathways model of problem and pathological gambling. Addiction, 97(5), 487–499. 12033650 10.1046/j.1360-0443.2002.00015.x

[mpr1865-bib-0005] Boffo, M. , Smits, R. , Salmon, J. P. , Cowie, M. E. , de Jong, D. T. H. A. , Salemink, E. , … Wiers, R. W. (2018). Luck, come here! Automatic approach tendencies toward gambling cues in moderate‐ to high‐risk gamblers. Addiction (Abingdon, England), 113(2), 289–298. 10.1111/add.14071 29055971

[mpr1865-bib-0006] Boren, T. , & Ramey, J. (2000). Thinking aloud: Reconciling theory and practice. IEEE Transactions on Professional Communication, 43(3), 261–278. 10.1109/47.867942

[mpr1865-bib-0007] Calado, F. , & Griffiths, M. D. (2016). Problem gambling worldwide: An update and systematic review of empirical research (2000–2015). Journal of Behavioral Addictions, 5(4), 592–613. 27784180 10.1556/2006.5.2016.073 PMC5370365

[mpr1865-bib-0008] Caler, K. , Garcia, J. , & Nower, L. (2016). Assessing problem gambling: A review of classic and specialized measures. Current Addiction Reports, 3(4), 437–444. 10.1007/s40429-016-0118-7

[mpr1865-bib-0009] Chamberlain, S. R. , Stochl, J. , Redden, S. A. , Odlaug, B. L. , & Grant, J. E. (2017). Latent class analysis of gambling subtypes and impulsive/compulsive associations: Time to rethink diagnostic boundaries for gambling disorder? Addictive Behaviors, 72, 79–85. 10.1016/j.addbeh.2017.03.020 28384607 PMC5457805

[mpr1865-bib-0010] Cowlishaw, S. , Merkouris, S. , Dowling, N. , Anderson, C. , Jackson, A. , & Thomas, S. (2012). Psychological therapies for pathological and problem gambling. Cochrane Depression, Anxiety and Neurosis Group, 11, CD008937. 10.1002/14651858.CD008937.pub2 PMC11955261 23152266

[mpr1865-bib-0011] De Vet, H. C. , Terwee, C. B. , Mokkink, L. B. , & Knol, D. L. (2011). Development of a measurement instrument. In Measurement in medicine: A practical guide (pp. 30–64). Cambridge, UK: Cambridge University Press.

[mpr1865-bib-0012] Denis, C. , Fatséas, M. , & Auriacombe, M. (2012). Analyses related to the development of DSM‐5 criteria for substance use related disorders: 3. An assessment of pathological gambling criteria. Drug and Alcohol Dependence, 122(1–2), 22–27. 10.1016/j.drugalcdep.2011.09.006 21962725

[mpr1865-bib-0013] Dowling, N. , Merkouris, S. , Manning, V. , Volberg, R. , Lee, S. , Rodda, S. , & Lubman, D. (2017). Screening for problem gambling within mental health services: A comparison of the classification accuracy of brief instruments. Addiction, 113(6), 1088–1104. 10.1111/add.14150 29274182

[mpr1865-bib-0014] Ericsson, K. A. , & Simon, H. A. (1980). Verbal reports as data. Psychological Review, 87(3), 215–251. 10.1037/0033-295X.87.3.215

[mpr1865-bib-0015] Ferris, J. , & Wynne, H. (2001). The Canadian Problem Gambling Index: Final report. Ottawa, Canada: Canadian Centre on Substance Abuse.

[mpr1865-bib-0016] Gehlbach, H. , & Brinkworth, M. E. (2011). Measure twice, cut down error: A process for enhancing the validity of survey scales. Review of General Psychology, 15(4), 380–387.

[mpr1865-bib-0017] Guyatt, G. , Oxman, A. , Kunz, R. , Atkins, D. , Brozek, J. , Vist, G. , … Schünemann, H. (2011). GRADE guidelines: 2. Framing the question and deciding on important outcomes. Journal of Clinical Epidemiology, 64(4), 395–400. 10.1016/j.jclinepi.2010.09.012 21194891

[mpr1865-bib-0018] Hildebrand, M. (2015). The psychometric properties of the Drug Use Disorders Identification Test (DUDIT): A review of recent research. Journal of Substance Abuse Treatment, 53, 52–59. 10.1016/j.jsat.2015.01.008 25682718

[mpr1865-bib-0019] Hodgins, D. C. (2004). Using the NORC DSM screen for gambling problems as an outcome measure for pathological gambling: Psychometric evaluation. Addictive Behaviors, 29(8), 1685–1690. 10.1016/j.addbeh.2004.03.017 15451138

[mpr1865-bib-0020] Hodgins, D. (2014). Time line follow back method: Retrospective report of gambling behaviour (Unpublished manuscript).

[mpr1865-bib-0021] Hodgins, D. C. , & Makarchuk, K. (2003). Trusting problem gamblers: Reliability and validity of self‐reported gambling behavior. Psychology of Addictive Behaviors, 17(3), 244–248. 10.1037/0893-164X.17.3.244 14498819

[mpr1865-bib-0022] Hollander, E. , Pallanti, S. , Allen, A. , Sood, E. , & Rossi, N. B. (2005). Does sustained‐release lithium reduce impulsive gambling and affective instability versus placebo in pathological gamblers with bipolar spectrum disorders?. American Journal of Psychiatry, 162(1), 137–145. 10.1176/appi.ajp.162.1.137 15625212

[mpr1865-bib-0023] Kuliś, D. , Whittaker, C. , Greimel, E. , Bottomley, A. , & Koller, M. (2017). Reviewing back translation reports of questionnaires: The EORTC conceptual framework and experience. Expert Review of Pharmacoeconomics & Outcomes Research, 17(6), 523–530. 10.1080/14737167.2017.1384316 28974101

[mpr1865-bib-0024] Lesieur, H. R. , & Blume, S. B. (1987). The South Oaks gambling screen (SOGS): A new instrument for the identification of pathological gamblers. The American Journal of Psychiatry, 144(9), 1184. 3631315 10.1176/ajp.144.9.1184

[mpr1865-bib-0025] Molander, O. , Månsson, V. , Sundqvist, K. , Wennberg, P. , Volberg, R. , & Berman, A. H. (2018). Development and psychometric evaluation of the Gambling Disorders Identification Test (GDIT)—An ongoing study . Alberta Gambling Research Institute's 17th Annual Conference Banff, Alberta, Canada.

[mpr1865-bib-0026] Molander, O. , Volberg, R. , Sundqvist, K. , Wennberg, P. , Månsson, V. , & Berman, A. H. (2019). Development of the gambling disorder identification test (GDIT): Protocol for a Delphi method study. JMIR Research Protocols, 8(1), e12006. 10.2196/12006 30622097 PMC6329424

[mpr1865-bib-0027] Neighbors, C. , Lostutter, T. , Larimer, M. , & Takushi, R. (2002). Measuring gambling outcomes among college students. Journal of Gambling Studies, 18(4), 339–360. 10.1023/A:1021013132430 12514914 PMC1797803

[mpr1865-bib-0028] Otto, J. L. , Smolenski, D. J. , Garvey Wilson, A. L. , Evatt, D. P. , Campbell, M. S. , Beech, E. H. , … Belsher, B. E. (2020). A systematic review evaluating screening instruments for gambling disorder finds lack of adequate evidence. Journal of Clinical Epidemiology, 120, P86–P93. 10.1016/j.jclinepi.2019.12.022 31917356

[mpr1865-bib-0029] Pallesen, S. , Mitsem, M. , Kvale, G. , Johnsen, B.‐H. , & Molde, H. (2005). Outcome of psychological treatments of pathological gambling: A review and meta‐analysis. Addiction, 100(10), 1412–1422. 10.1111/j.1360-0443.2005.01204.x 16185203

[mpr1865-bib-0030] Petry, N. M. , Ginley, M. K. , & Rash, C. J. (2017). A systematic review of treatments for problem gambling. Psychology of Addictive Behaviors, 31(8), 951–961. 10.1037/adb0000290 28639817 PMC5714688

[mpr1865-bib-0031] Pickering, D. , Keen, B. , Entwistle, G. , & Blaszczynski, A. (2017). Measuring treatment outcomes in gambling disorders: A systematic review. Addiction (Abingdon, England), 113(3), 411–426. 10.1111/add.13968 PMC5836978 28891116

[mpr1865-bib-0032] R Core Team . (2018). R: A language and environment for statistical computing. Vienna, Austria: R Foundation for Statistical Computing. Retrieved from https://www.R-project.org/

[mpr1865-bib-0033] Reinert, D. F. , & Allen, J. P. (2002). The Alcohol Use Disorders Identification Test (AUDIT): A review of recent research. Alcoholism: Clinical and Experimental Research, 26(2), 272–279. 10.1111/j.1530-0277.2002.tb02534.x 11964568

[mpr1865-bib-0034] Rockloff, M. (2012). Validation of the consumption screen for problem gambling (CSPG). Journal of Gambling Studies, 28(2), 207–216. 10.1007/s10899-011-9260-2 21830133

[mpr1865-bib-0035] Saunders, J. B. , Aasland, O. G. , Babor, T. F. , de La Fuente, J. R. , & Grant, M. (1993). Development of the alcohol use disorders identification test (AUDIT): WHO collaborative project on early detection of persons with harmful alcohol consumption–II. Addiction (Abingdon, England), 88(6), 791. 10.1111/j.1360-0443.1993.tb02093.x 8329970

[mpr1865-bib-0036] Shaffer, H. , LaBrie, R. , Scanlan, K. , & Cummings, T. (1994). Pathological gambling among adolescents: Massachusetts gambling screen (MAGS). Journal of Gambling Studies, 10(4), 339–362. 10.1007/BF02104901 24234969

[mpr1865-bib-0037] Shorter, G. W. , Heather, N. , Bray, J. W. , Berman, A. H. , Giles, E. L. , O'Donnell, A. J. , … Newbury‐Birch, D. (2019). Prioritization of outcomes in efficacy and effectiveness of alcohol brief intervention trials: International multi‐stakeholder e‐Delphi consensus study to inform a core outcome set. Journal of Studies on Alcohol and Drugs, 80(3), 299–309. 10.15288/jsad.2019.80.299 31250794

[mpr1865-bib-0038] Sobell, L., C. , & Sobell, M., B. (1996). Timeline follow‐back user's guide: A calendar method assessing alcohol and drug abuse. Toronto, ON: Addiction Research Foundation.

[mpr1865-bib-0039] Stinchfield, R. , McCready, J. , Turner, N. , Jimenez‐Murcia, S. , Petry, N. , Grant, J. , … Winters, K. (2016). Reliability, validity, and classification accuracy of the DSM‐ 5 diagnostic criteria for gambling disorder and comparison to DSM‐ IV. Journal of Gambling Studies, 32(3), 905–922. 10.1007/s10899-015-9573-7 26408026 PMC4993799

[mpr1865-bib-0040] Suck Won Kim, J. E. , Grant, M. N. , Potenza, C. , Blanco, E. , & Hollander, E. (2009). The Gambling Symptom Assessment Scale (G‐ SAS): A reliability and validity study. Psychiatry Research, 166(1), 76–84. 10.1016/j.psychres.2007.11.008 19200607 PMC3641525

[mpr1865-bib-0041] Tolchard, B. , & Battersby, M. (2010). The Victorian gambling screen: Reliability and validation in a clinical population. Journal of Gambling Studies, 26(4), 623–638. 10.1007/s10899-009-9172-6 20035440

[mpr1865-bib-0042] Volberg, R. A. , & Williams, R. J. (2011). *Developing a brief problem gambling screen using clinically validated samples of at‐risk, problem and pathological gamblers* (Report to the Alberta Gaming Research Institute).

[mpr1865-bib-0043] Walker, M. , Toneatto, T. , Potenza, M. N. , Petry, N. , Ladouceur, R. , Hodgins, D. C. , … Blaszczynski, A. (2006). A framework for reporting outcomes in problem gambling treatment research: The Banff, Alberta consensus. Addiction, 101(4), 504–511. 10.1111/j.1360-0443.2005.01341.x 16548930

[mpr1865-bib-0044] Weinstock, J. , Whelan, J. P. , & Meyers, A. W. (2004). Behavioral assessment of gambling: An application of the timeline followback method. Psychological Assessment, 16(1), 72–80. 10.1037/1040-3590.16.1.72 15023094

[mpr1865-bib-0045] Williams, R. J. , & Volberg, R. A. (2013). The classification accuracy of four problem gambling assessment instruments in population research. International Gambling Studies, 40, 15–28. 10.1080/14459795.2013.839731

[mpr1865-bib-0046] Williams, R. J. , Volberg, R. A. , & Stevens, R. M. G. (2012). *The population prevalence of problem gambling: Methodological influences, standardized rates, jurisdictional differences, and worldwide trends* [technical report]. Ontario Problem Gambling Research Centre. Retrieved from https://opus.uleth.ca/handle/10133/3068

[mpr1865-bib-0047] Williams, R. J. , Volberg, R. A. , Stevens, R. M. , Williams, L. A. , & Arthur, J. N. (2017). The definition, dimensionalization, and assessment of gambling participation. Canadian Consortium for Gambling Research.

[mpr1865-bib-0048] Wood, R. T. , & Williams, R. J. (2007). How much money do you spend on gambling?' the comparative validity of question wordings used to assess gambling expenditure. International Journal of Social Research Methodology, 10(1), 63–77. 10.1080/13645570701211209

