# Supplementary figures and images for: Development of the Gambling Disorder Identification Test: Results from an international Delphi and consensus process
Source: Int J Methods Psychiatr Res. 2020 Nov 21;30(2):e1865. doi: 10.1002/mpr.1865 (PMC8170582; doi:10.1002/mpr.1865)

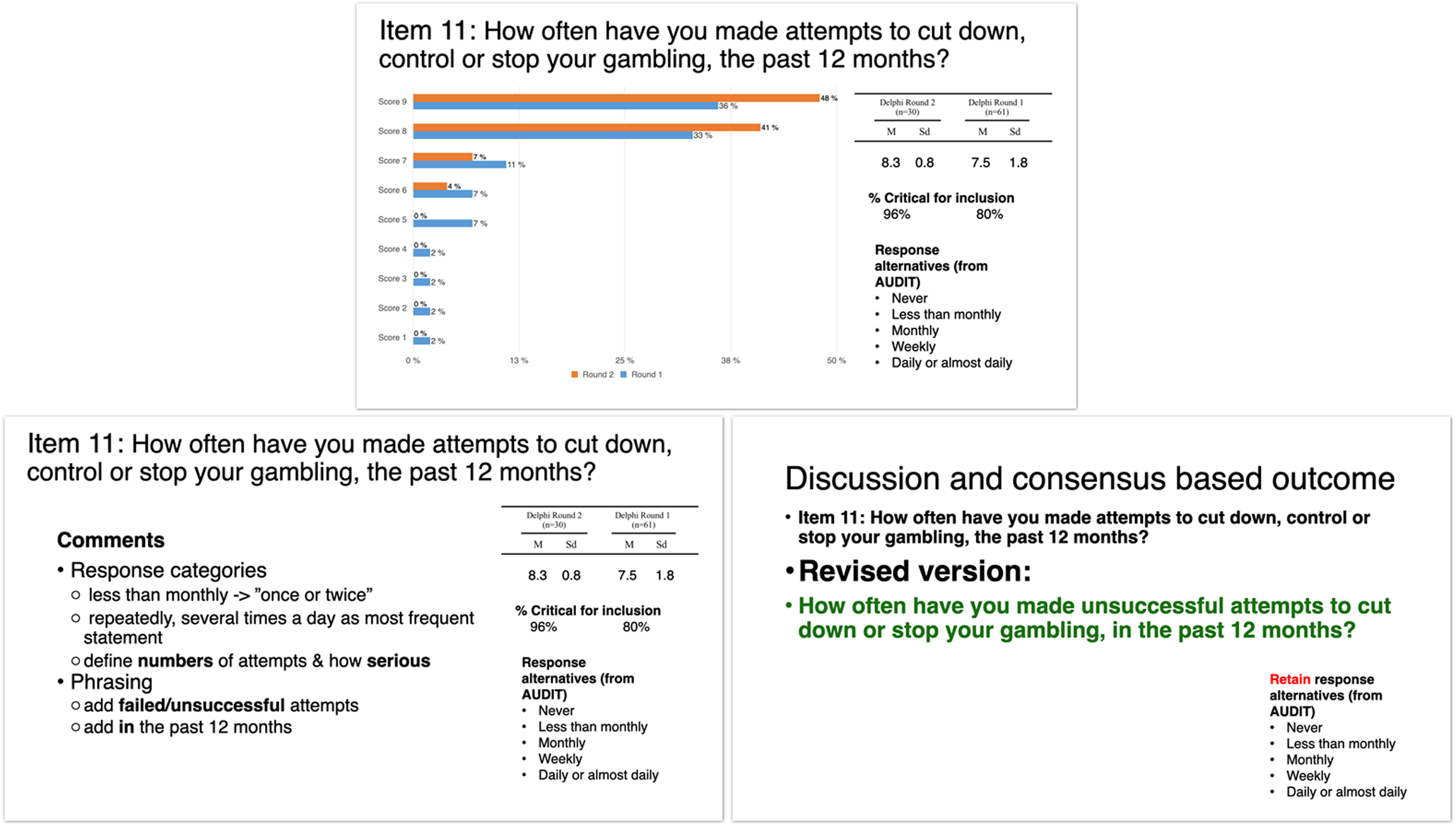

Supplement: Supplementary file 1 — Supplementary Material [file MPR-30-e1865-s001.tif]
